# Differences in clinical characteristics and liver injury between patients diagnosed with the Omicron subvariant BA.5.2 and the prototype of SARS-CoV-2: a single center retrospective study

**DOI:** 10.1186/s12876-023-02907-z

**Published:** 2023-08-08

**Authors:** Jie Li, Qing Zhang, Chao Xu, Yan Zhang, Yueyue Lu, Minghua Ai, Xiaoping Tan

**Affiliations:** 1 Department of Gastroenterology, Jingzhou of Hubei Province, First Hospital of Yangtze University, Jingzhou, China; 2 Digestive Disease Research Institution of Yangtze University, Jingzhou, China; 3 Clinical medical college, Jingzhou of Hubei Province, Yangtze University, Jingzhou, China

**Keywords:** COVID-19, SARS-CoV-2, Omicron subvariant BA.5.2, Liver injury

## Abstract

**Background:**

The purpose of this study was to investigate the differences between the clinical characteristics and the factors influencing liver injury in patients with the Omicron subvariant BA.5.2 (Omicron BA.5.2) and the prototype of severe acute respiratory syndrome coronavirus 2 (SARS-CoV-2).

**Methods:**

Between December 30, 2019 and November 30, 2022, 157 patients infected with the SARS-CoV-2 prototype and 199 patients infected with the Omicron BA.5.2 were included in this case-control, single-center, retrospective study. Differences in clinical characteristics and liver injury between the Omicron BA.5.2 patients and the prototype patients were subsequently analyzed.

**Results:**

None of the Omicron BA.5.2 patients reached the critical state, and showed relatively milder symptoms including fever, cough, headache, muscle soreness, nausea or vomiting, diarrhea, anorexia and hypoxia. The Omicron BA.5.2 had a lower effect on body temperature (T), white blood cell (WBC) count, hematocrit (HCT), C-reactive protein (CRP) level, D-dimer, finger pulse oxygen saturation (SpO_2_) and lung lesions. The differences in liver injury between the two groups were related to the severity of the disease, T, blood oxygen levels, albumin (ALB), CRP, and medication usage. Gender, body mass index, and CRP levels influenced liver damage in the Omicron BA.5.2 patients. In particular, CRP was an independent risk factor for liver injury. Because the severity of liver function damage was considerably low, only a small number of Omicron BA.5.2 patients required liver-protective treatment.

**Conclusion:**

Liver injury is expected in the COVID-19 patients. The Omicron BA.5.2 patients showed milder symptoms of liver injury than the prototype patients. However, dynamic monitoring of liver function is warranted, especially for individuals presenting with elevated levels of CRP.

## Introduction

The first case of severe acute respiratory syndrome coronavirus 2 (SARS-CoV-2) infection was first reported in Wuhan, China (December 2019), and the resulting condition was officially named coronavirus infectious disease 2019 (COVID-19) by the World Health Organization (WHO) (January 2020). COVID-19 has posed a serious threat to global public health [1–2]. As of November 8, 2022, more than 638 million confirmed cases and 6.8 million COVID-19-related deaths have been reported [1]. The increasing accumulation of mutations in the virus genome has resulted in the formation of new lineages. Five variants of concerns (VOCs) have been reported thus far, including Alpha (B.1.1.7) (prototype), Beta (B.1.351), Gamma (P.1), Delta (B.1.617.2), and Omicron (B.1.1.529) [3–6].

The SARS-CoV-2 variant named Omicron (B.1.1.529) was first detected in South Africa on November 14, 2021, as the fifth VOC [7]. Numerous Omicron variants have subsequently emerged and spread globally, with these variants being classified into five primary lineages: BA.1, BA.2, BA.3, BA.4, and BA.5. Some of the sublineages include BA.1.1, BA.2.12.1, BA.2.11, BA.2.75, BA.4.6, BA.5.1, and BA.5.2. All Omicron lineages show multiple mutations, of which 31–37 mutations were observed in spike proteins, leading to relatively higher rates of infection and morbidity than previous VOCs [3, 8–10]. The first case of Omicron subvariant BA.5.2 (Omicron BA.5.2), which was accidentally imported into Beijing, China, was discovered on July 4, 2022. Since then, this SARS-CoV-2 variant rapidly spread within the country and has reportedly become the dominant strain in some cities [11].

According to existing research, SARS-CoV-2 is the most well-known etiological agent for substantial respiratory pathology; furthermore, it may lead to several extrapulmonary manifestations, including gastrointestinal and liver injury, acute kidney injury, and neurological and psychiatric illnesses. Among these, liver injury is relatively the most common [12–14]. COVID-19-associated liver injury occurs as a result of the cumulative effects of multiple factors. SARS-CoV-2 RNA expression has been detected in liver tissue, potentially causing hepatocellular lesions directly. Moreover, liver injury may be associated with drug-induced liver injury, hypoxic reperfusion, immune stress, and inflammatory factor storms [15–16]. The data of 12 studies showed that the pooled prevalence of liver injury, the increased alanine aminotransferase (ALT), increased aspartate aminotransferase (AST), and decreased albumin (ALB) levels were 19%, 18%, 21%, and 6%, respectively [17]. Additionally, liver injury is more prevalent in severe cases than in mild cases of COVID-19 [18]. The Omicron variants reported here may not have been as severe as the previous episodes; however, additional evidence is needed to determine whether the Omicron variants are relatively more benign [10]. The characteristics of the impact of the earliest COVID-19 (prototype) patients on liver function have been analyzed in the past [19]. In this study, we aim to further analyze differences in clinical characteristics and liver injury between patients diagnosed with the Omicron BA.5.2 and the prototype.

## Methods

### Study design and patients

In the single-center retrospective study, patients admitted to the First Hospital of Yangtze University and infected with the prototype and Omicron BA.5.2 were included; thus, patients aged < 18 years, with serious underlying diseases, and pregnant women were excluded. The COVID-19 diagnosis was established based on the New Coronavirus Pneumonia Prevention and Control Program (9th edition) published by the National Health Commission of China and the interim guidance from the WHO [19–20]. A positive COVID-19 PCR test confirms diagnosis, leading to hospitalization and isolation treatment according to local policies. Nasopharyngeal swab samples from 356 patients who were tested at the Jingzhou Centers for Disease Control laboratory genetic sequencing of the virus were Omicron BA.5.2 and prototype from December 30, 2019, to November 30, 2022.

### Data collection

The general information and clinical symptoms of all cases were collected. General information included gender, age, body weight, height, history of smoking and vaccination, and comorbidities—e.g., chronic obstructive pulmonary disease, hypertension, coronary heart disease and/or diabetes, viral hepatitis, and fatty liver. Clinical symptoms involved body temperature (T), finger pulse oxygen saturation (SpO_2_), respiratory symptoms (e.g., fevers, cough, and sore throat, among others), and digestive symptoms (e.g., diarrhea and anorexia, among others). Laboratory tests included routine blood tests (white blood cell [WBC], lymphocyte [LY], and platelet [PLT] count hematocrit [HCT]), coagulation function (international normalized ratio [INR] and plasma prothrombin time[PT]), liver function (alanine transaminase [ALT], aspartate aminotransferase [AST], prealbumin [PA], albumin [ALB], total bilirubin [TB], alkaline phosphatase [ALP], g-glutamyl transpeptidase [GGT], lactate dehydrogenase [LDH], and cholinesterase [CHE]), C-reactive protein (CRP), D-dimer, IL-6, computed tomography (CT) imaging presentations, therapeutic drugs, and disease prognosis. Reverse transcription-quantitative polymerase chain reaction (RT-qPCR) was used to detect SARS-CoV-2 in samples collected via nasopharyngeal swabs. According to the central laboratory report specification prepared by the authors of the present study, the upper limits of normal (ULN) of ALT, AST, ALP, GGT, and LDH were 40, 42, 128, 50, and 240 U/L, respectively. In addition, the ULN of TB was 20.4 µmol/L, the lower limits of normal (LLN) of ALB was 35 g/L. Liver injury is defined as any exceedance of the ULN for liver function parameters including ALT, AST, ALP, GGT, LDH, and TB, or below the LLN for ALB.

### Statistical analyses

Statistical analyses for all data were performed using the SPSS software version 22.0 (IBM Inc., Chicago, IL). Categorical variables were presented as numbers (percentages) and were analyzed using the Chi-Squared test or Fisher exact test. Measurement data were presented as mean ± standard deviation and were analyzed using the Student’s t-test for intergroup comparisons. Multiple factors were analyzed using logistic regression. The histograms were drawn by GraphPad Prism 8. Furthermore, a two-sided P < 0.05 indicated statistical significance.

## Results

### Epidemiological and clinical characteristics of the Omicron BA.5.2 and prototype patients

The Omicron BA.5.2 patients (n = 199) and prototype patients (n = 157) from December 30, 2019, to November 30, 2022, were included. The demographic and clinical characteristics are shown in Table 1. There were no significant differences between the two groups in terms of gender, age, smoking history, and comorbidities (coronary heart disease, hypertension, pulmonary disease, and liver disease) (*P* > 0.05) .

**Table 1:** Epidemiological and clinical characteristics of the Omicron BA.5.2 and prototype patients

	Prototype	Omicron BA.5.2	t/χ^2^	*P*
N	157	199		
Gender				
male	81 (51.6%)	110 (55.3%)	0.479	0.489
female	76 (48.4%)	89 (44.7%)		
Age (years)	51.49 ± 17.39	51.49 ± 13.51	0.002	0.998
Smoking history	17 (10.8%)	34 (17.1%)	2.800	0.090
Comorbidity				
Hypertension	31 (19.7%)	44 (22.1%)	0.295	0.587
Coronary heart disease	4 (2.5%)	11 (5.5%)	1.931	0.165
COPD	1 (0.6%)	3 (1.5%)	0.599	0.439
Pulmonary tuberculosis	1 (0.6%)	3 (1.5%)	0.599	0.439
Chronic bronchitis	3 (1.9%)	3 (1.5%)	0.086	0.769
Viral hepatitis	4 (2.5%)	3 (1.5%)	0.493	0.483
Fatty liver	4 (2.5%)	2 (1.0%)	1.261	0.262
Signs and symptoms				
asymptomatic	2 (1.0%)	62 (31.2%)	53.140	< 0.001
Fever	118 (75.2%)	85 (39.2%)	37.700	< 0.001
Cough	94 (59.9%)	78 (39.2%)	15.030	< 0.001
Fatigue	39 (24.8%)	8 (4.0%)	33.200	< 0.001
Chest tightness/dyspnea	28 (17.8%)	2 (1.0%)	32.210	< 0.001
Sore throat	13 (8.3%)	26 (13.1%)	2.060	0.001
Headache	6 (3.8%)	21 (10.6%)	6.264	0.012
Muscle soreness	22 (14.0%)	9 (4.5%)	9.092	0.002
Nausea or vomiting	7 (4.5%)	5 (2.5%)	1.020	0.312
Diarrhea	16 (10.2%)	4 (2.0%)	5.651	0.017
Anorexia	28 (17.8%)	5 (2.5%)	10.070	0.001
Type of the novel coronavirus				
Mild/Common	112 (71.3%)	199 (100%)	65.290	< 0.001
Severe/Critical	45 (28.7%)	0 (0.0%)		
T (℃)	37.8 ± 0.93	37.37 ± 0.98	3.948	< 0.001
Mortality	11 (7.0%)	0 (0.0%)	14.390	< 0.001

COPD = chronic obstructive pulmonary disease, T = temperature

The proportion of prototype patients showing symptoms of fever (75.2% vs. 39.2%, *P* < 0.001), cough (59.9% vs. 39.2%, *P* < 0.001), fatigue (24.8% vs. 4.0%, P < 0.001), chest tightness/dyspnea (17.8% vs. 1.0%, *P* < 0.001), muscle soreness (14.0% vs. 4.5%, *P* = 0.002), diarrhea (10.2% vs. 2.0%, *P* = 0.017), and anorexia (17.8% vs. 2.5%, *P* = 0.001) was significantly higher than the Omicron BA.5.2 patients. However, a higher proportion of the Omicron BA.5.2 patients were asymptomatic (31.2% vs. 1.0%, *P* < 0.001) and more frequently presented with symptoms of headache (10.6% vs. 3.8%, *P* = 0.012) and sore throat (13.1% vs. 8.3%, *P* = 0.001) than the prototype patients. In total, among the prototype patients, there were 45 severe or critical cases and 11 deaths. However, none of the Omicron BA.5.2 patients were in a critical condition or had died(*P* < 0.001).

### Laboratory and radiological characteristics of the Omicron BA.5.2 and prototype patients

The prototype patients (92.50 ± 15.00) exhibited significantly lower SpO_2_ levels than the Omicron BA.5.2 patients (97.51 ± 1.48) (X^2^ = 4.403, *P* < 0.001). Regarding pulmonary CT examination, higher ratios of ground-glass opacity (19.1% vs. 6.5%), patchy shadowing (68.2% vs. 34.2%), and consolidation (11.5% vs. 0.0%) were observed in the prototype patients compared to those with the Omicron BA.5.2 patients (X^2^ = 142.600, *P* < 0.001). However, none of the 118 Omicron BA.5.2 patients (59.3%) exhibited signs of pneumonia. In terms of routine blood tests, abnormalities in WBC counts, PLT, and HCT were less common in Omicron BA.5.2 patients than in the prototype patients (*P* < 0.001), though the decrease in lymphocyte counts was higher in the Omicron BA.5.2 patients (*P* < 0.001). 73 Omicron BA.5.2 patients (36.7%) showed increased CRP levels and 8 patients (4.0%) had increased D-dimer, both lower than the prototype patients (*P* < 0.001). In terms of coagulation function, INR (1.00 ± 0.15 vs. 0.91 ± 0.09) and PT (11.20 ± 1.68 vs. 10.46 ± 0.82) in the prototype patients were higher compared to Omicron BA.5.2 patients (*P* < 0.001). In terms of liver function, the prototype patients showed significantly higher rates of abnormalities in PA (81.5% vs. 16.6%), ALB (41.4% vs. 0.5%), TB (33.8% vs. 6.5%), ALT (50.3% vs. 11.1%), AST (37.6% vs. 9.5%), GGT (38.9% vs. 15.1%), ALP (8.3% vs. 1.5%), LDH (45.9% vs. 1.5%) and CHE (14.6% vs. 0.0%) than the Omicron BA.5.2 patients (*P* < 0.05) (Table 2).

**Table 2:** Laboratory and radiological characteristics of the Omicron BA.5.2 and prototype patients

	Prototype	Omicron BA.5.2	t /χ^2^	*P*
N	157	199		
SpO_2_ (%)	92.50 ± 15.00	97.51 ± 1.48	4.403	< 0.001
CT			142.600	< 0.001
No pneumonia	2 (1.3%)	118 (59.3%)		
Ground-glass opacity	30 (19.1%)	13 (6.5%)		
Local/Bilateral patchy shadowing	107 (68.2%)	68 (34.2%)		
consolidation	18 (11.5%)	0 (0.0%)		
White-cell count (×10^9^·L^-1^)			19.080	< 0.001
≥ 10	19 (12.1%)	3 (1.5%)		
≥ 4, < 10	125 (79.6%)	167 (83.9%)		
< 4	13 (8.3%)	29 (14.6%)		
Lymphocyte count (×10^9^·L^-1^)			19.980	< 0.001
≥ 4	2 (1.3%)	0 (0.0%)		
≥ 0.8, <4	97 (61.8%)	80 (40.2%)		
< 0.8	58 (36.9%)	119 (59.8%)		
Platelet count (×10^9^·L^-1^)			39.740	< 0.001
≥ 300	4 (2.5%)	5 (2.5%)		
≥ 100, < 300	112 (71.3%)	187 (94.0%)		
< 100	41 (26.1%)	7 (3.5%)		
Hematocrit (%)				
(< 40 (male), < 35 (female))	98 (62.4%)	15 (7.5%)	112.000	< 0.001
CRP (> 8 mg.L^-1^)	111 (70.7%)	73 (36.7%)	40.670	< 0.001
D-dimer (> 0.55 mg.L^-1^)	104 (66.2%)	8 (4.0%)	157.600	< 0.001
PT (second)	11.20 ± 1.68	10.46 ± 0.82	7.234	< 0.001
INR	1.00 ± 0.15	0.91 ± 0.09	4.727	< 0.001
Liver function				
PA (< 200 mg.L^-1^)	128 (81.5%)	33 (16.6%)	114.800	< 0.001
ALB (< 35 g.L^-1^)	65 (41.4%)	1 (0.5%)	100.100	< 0.001
TB (> 20.4 umol.L^-1^)	53 (33.8%)	13 (6.5%)	42.220	< 0.001
ALT (> 40 U.L^-1^)	79 (50.3%)	22 (11.1%)	88.610	< 0.001
AST (> 42 U.L^-1^)	59 (37.6%)	19 (9.5%)	40.770	< 0.001
LDH (> 240 U.L^-1^)	72 (45.9%)	3 (1.5%)	64.250	< 0.001
GGT (> 50 U.L^-1^)	61 (38.9%)	30 (15.1%)	10.490	0.001
ALP (> 128 U.L^-1^)	13 (8.3%)	3 (1.5%)	8.635	0.003
CHE (> 3700 U.L^-1^)	23 (14.6%)	0 (0.0%)	17.500	< 0.001

SpO2 = finger pulse oxygen saturation, CT = computed tomography, PT = plasma prothrombin time, INR = International Normalized Ratio, PA = prealbumin, ALB = albumin, TB = total bilirubin, ALT = alanine transaminase, AST = aspartate aminotransferase, LDH = lactate dehydrogenase, GGT = γ-glutamyl transpeptidase, ALP = alkaline phosphatase, CHE = cholinesterase

### Baseline level of liver function in the Omicron BA.5.2 and prototype patients

Among the 157 prototype patients, the baseline levels of ALT, AST, TB, ALB, LDH, GGT, PA, and CHE were 76.14 ± 108.40 U/L, 46.22 ± 35.75 U/L, 19.74 ± 15.35 µmol/L, 36.70 ± 4.64 g/L, 251.10 ± 154.70 U/L, 88.24 ± 150.00 U/L, 137.50 ± 68.45 mg/L, and 5292.00 ± 2083.00 U/L, respectively. Among the 199 Omicron BA.5.2 patients, the baseline levels of ALT, AST, TB, ALB, LDH, GGT, PA, and CHE were 22.27 ± 21.95 U/L, 28.88 ± 21.06 U/L, 11.95 ± 5.07 µmol/L, 42.97 ± 2.24 g/L, 159.20 ± 31.91 U/L, 34.91 ± 38.54 U/L, 237.20 ± 68.45 mg/L, and 8451.00 ± 1793.00 U/L, respectively. The Omicron BA.5.2 patients exhibited significantly lower levels of ALT (t = 6.839, *P* < 0.001), AST (t = 5.696, *P* < 0.001), TB (t = 6.707, *P* < 0.001), GGT (t = 4.809, *P* < 0.001), and LDH (t = 6.301, *P* < 0.001). They also had significantly higher levels of ALB (t = 17.730, *P* < 0.001), PA (t = 12.540, *P* < 0.001), and CHE (t = 4.809, *P* < 0.001). All these differences were statistically significant. However, no intergroup differences were noted in terms of the ALP levels (t = 1.436, *P* = 0.152) (Table 3).

**Table 3:** Baseline level of liver function in the Omicron BA.5.2 and prototype patients

	Prototype	Omicron BA.5.2	t /χ2	*P*
N	157	199		
PA (mg.L^-1^)	137.50 ± 68.45	237.20 ± 68.45	12.540	< 0.001
ALB (g·L^-1^ )	36.70 ± 4.64	42.97 ± 2.24	17.730	< 0.001
TB (umol.L^-1^)	19.74 ± 15.35	11.95 ± 5.07	6.707	< 0.001
AST (U.L^-1^)	46.22 ± 35.75	28.88 ± 21.06	5.696	< 0.001
ALT (U.L^-1^)	76.14 ± 108.40	22.27 ± 21.95	6.839	< 0.001
GGT (U.L^-1^)	88.24 ± 150.00	34.91 ± 38.54	4.809	< 0.001
CHE (U.L^-1^)	5292.00 ± 2083.00	8451.00 ± 1793.00	10.460	< 0.001
ALP (U.L^-1^)	82.20 ± 78.05	73.91 ± 20.56	1.436	0.152
LDH (U.L^-1^)	251.10 ± 154.70	159.20 ± 31.91	6.301	< 0.001

PA = prealbumin, ALB = albumin, TB = total bilirubin, AST = aspartate aminotransferase, ALT = alanine transaminase, GGT = γ-glutamyl transpeptidase, CHE = cholinesterase, ALP = alkaline phosphatase, LDH = lactate dehydrogenase

**Fig. 1:**
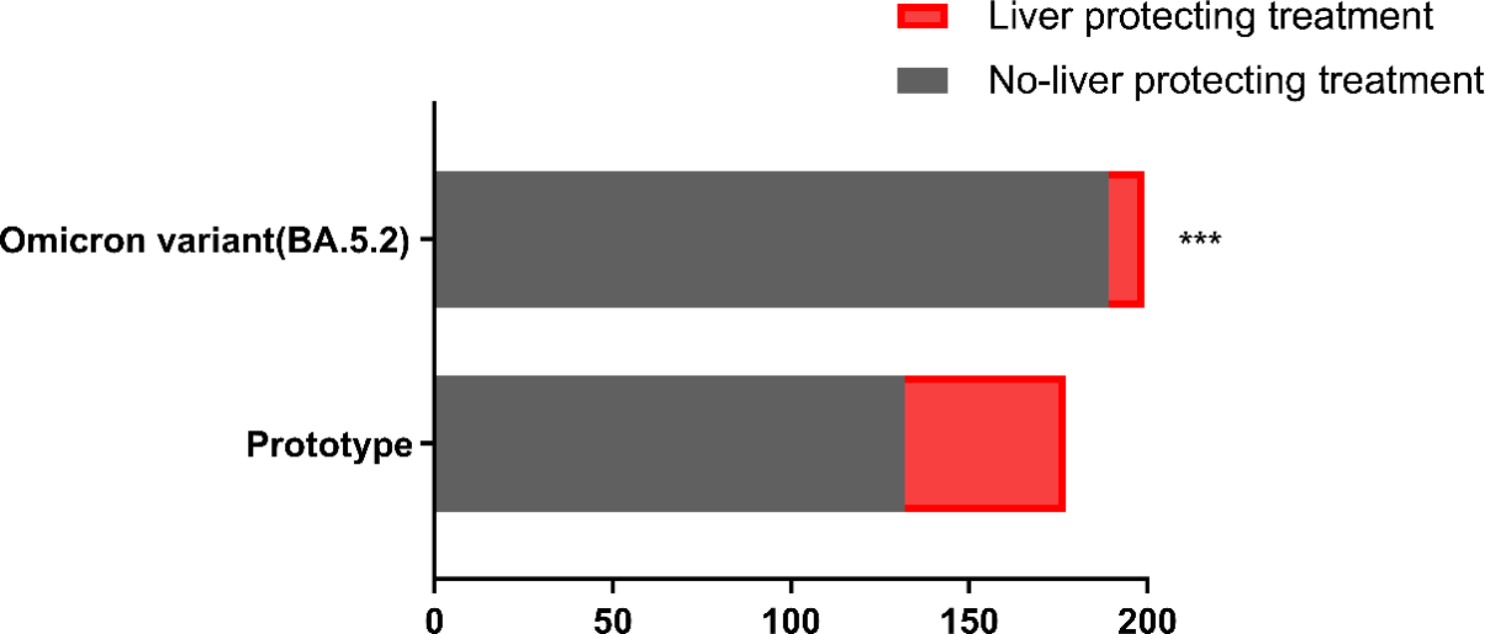
The number of patients infected with prototype and Omicron BA.5.2 using the hepatoprotective drugs(^***^P < 0.001)

We further analyzed the extent of liver function impairment in both groups. The proportion of ALT ( t = 49.710, *P* < 0.001), AST (t = 39.770, *P* < 0.001), TB (t = 10.370, *P* < 0.001), GGT (t = 25.100, *P* < 0.001), and LDH (t = 26.860, *P* < 0.001) levels greater than 2ULN in the prototype patients was significantly higher than in the Omicron BA.5.2 patients. Additionally, the proportion of ALB levels (t = 98.900, *P* < 0.001) lower than 35 g/L in prototype patients was higher compared to the Omicron BA.5.2 patients (Table 4). A total of 45 the prototype patients (28.7%) and 10 the Omicron BA.5.2 patients (5.0%) were treated with hepatoprotective drugs (t = 31.210, *P* < 0.001) (Fig. 1).

**Table 4:** Comparison of abnormal liver function indexes in the Omicron BA5.2 and prototype patients

	Prototype	Omicron BA.5.2	t /χ^2^	*P*
ALT / ( U·L^-1^ )	129.80 ± 131.60	66.68 ± 39.68	2.208	0.030
1–2 ULN	34 (21.7%)	17 (8.5%)	12.300	< 0.001
> 2 ULN	45 (28.7%)	5 (2.5%)	49.710	< 0.001
AST / ( U·L^-1^ )	81.18 ± 40.54	53.11 ± 12.79	2.975	0.004
1–2 ULN	20 (12.7%)	16 (8.0%)	2.132	0.144
> 2 ULN	35 (22.3%)	3 (1.5%)	39.770	< 0.001
ALB / ( g·L^-1^ )	36.70 ± 4.64	42.97 ± 2.24	17.730	< 0.001
≥ 35	93 (59.2%)	199 (100.0%)	98.900	< 0.001
< 35	64 (40.7%)	0 (0.0%)	98.900	< 0.001
TB / ( umol·L^-1^ )	31.09 ± 19.25	25.25 ± 4.60	1.082	0.283
1–2 ULN	46 (29.3%)	13 (5.5%)	32.900	< 0.001
> 2 ULN	8 (5.1%)	0 (0.0%)	10.370	0.001
GGT/ ( U·L^-1^ )	184.70 ± 206.40	103.20 ± 63.14	2.110	0.038
1–2 ULN	25 (15.9%)	20 (10.1%)	2.742	0.098
> 2 ULN	36 (22.9%)	10 (5.0%)	25.100	< 0.001
LDH/ ( U·L^-1^ )	538.10 ± 1074.00	278.30 ± 28.68	0.416	0.679
1–2 ULN	52 (33.1%)	3 (1.5%)	67.150	< 0.001
> 2ULN	20 (12.7%%)	0 (0.0%)	26.860	< 0.001

ALT = alanine transaminase, ULN = upper limit of normal, AST = aspartate aminotransferase, ALB = albumin, TB = total bilirubin, GGT = γ-glutamyl transpeptidase, LDH = lactate dehydrogenase

### Influencing factors of abnormal liver function in the Omicron BA.5.2 and prototype patients

We discussed the probable reasons for the differences in liver damage between the Omicron BA.5.2 patients (n = 26) and the prototype patients (n = 77). No significant differences were noted in terms of gender (*P* = 0.106), age (*P* = 0.619), and comorbidities (*P* = 0.069). The Omicron BA.5.2 patients were vaccinated, and none of them were critically ill (*P* < 0.001). The SpO_2_ (*P* = 0.027) and ALB (*P* < 0.001) levels in the prototype patients were significantly lower than the Omicron BA.5.2 patients, and the temperature (*P* = 0.041) and CRP levels (*P* = 0.001) were significantly higher. However, D-dimer levels showed no significant difference (*P* = 0.161). Besides, the prototype patients received a wider variety of medications. Hormones (*P* < 0.001), antibiotics (*P* < 0.001), and chloroquine (*P* = 0.019) may be associated with liver damage, there was no difference in liver damage among patients using Chinese patent medicine (*P* = 0.115 ) (Table 5).

**Table 5:** Influencing factors of abnormal liver function in the Omicron BA5.2 and prototype patients

	Prototype	Omicron BA.5.2	t /χ^2^	*P*
N	77	26		
Gender			2.620	0.106
male	49 (63.6%)	21 (84.0%)		
female	28 (26.4%)	5 (16.0%)		
Age (years)	54.8 ± 16.64	53.0 ± 13.42	0.499	0.619
Vaccination History	0 (0.0%)	26 (100.0%)	103.000	< 0.001
Type of the novel coronavirus			20.740	< 0.001
Mild/Common	44 (57.1%)	26 (100.0%)		
Severe/Critical	43 (42.9%)	0 (0.0%)		
Complications	26 (33.8%)	14 (18.2%)	3.299	0.069
SpO_2_ (%)	91.56 ± 13.70	97.62 ± 1.65	2.242	0.027
T (℃)	37.93 ± 0.85	37.49 ± 1.20	2.069	0.041
ALB(mg·L^-1^ )	35.01 ± 4.71	42.11 ± 3.05	7.711	< 0.001
CRP(g·L^-1^ )	40.00 ± 57.21	16.76 ± 22.56	3.127	0.001
D-dimer(mg·L^-1^)	5.91 ± 10.22	0.40 ± 0.35	2.069	0.161
Therapeutic drugs				
Hormone	50 (64.9%)	0 (0.0%)	32.810	< 0.001
Antibiotic	70 (90.9%)	3 (11.5%)	59.320	< 0.001
Chinese patent medicine	62 (80.5%)	17 (65.4%)	2.491	0.115
Chloroquine	14 (18.2%)	0 (0.0%)	5.471	0.019

SpO2 = finger pulse oxygen saturation, T = temperature, ALB = albumin

### Influencing factors of abnormal liver function in the Omicron BA.5.2 patients

We analyzed influencing factors of normal (n = 173) and abnormal (n = 26) liver function in the Omicron BA.5.2 patients. Liver injury in those patients was significantly correlated with gender (*P* = 0.005), body mass index (BMI) (*P* = 0.020), and CRP levels (*P* < 0.001). However, no correlation was found with age, vaccination history, comorbidities, SpO_2_, T, Alb, WBC count, PT, INR, or therapeutic drugs. CRP was an independent risk factor for liver injury (Waldχ^2^ = 6.067 *P* < 0.05) (Table 6).

**Table 6:** Influencing factors of liver injury in the Omicron BA.5.2 patients

			Univariate analysis		Multivariate analysis	
	Normal group	Abnormal group	t/χ^2^	*P*	Waldχ^2^	*P*
N	173	26				
Male	89 (51.4%)	21 (80.8%)	7.862	0.005	0.000	0.999
Age (years)	51.25 ± 13.54	53.04 ± 13.43	0.627	0.531		
Vaccination History	171 (98.8%)	26 (100.0%)	0.304	0.589		
Classification						
Mild	102 (59.0%)	12 (46.2%)	1.515	0.281		
Common	71 (41.0%)	14 (53.8%)				
Comorbidities	43 (24.9%)	6 (23.1%)	0.039	0.844		
BMI	24.47 ± 3.93	26.70 ± 4.14	2.364	0.020	0.357	0.550
SpO_2_ (%)	97.44 ± 1.56	97.88 ± 1.21	1.384	0.168		
T (℃)	37.34 ± 0.95	37.47 ± 1.19	0.613	0.540		
ALB(g·L^-1^)	43.83 ± 1.87	43.72 ± 2.73	0.291	0.772		
WBC(×10^9^ L^-1^)	1.58 ± 1.50	4.79 ± 1.52	0.657	0.512		
CRP(mg·L^-1^)	9.02 ± 11.07	23.01 ± 31.12	4.377	0.000	6.067	0.010
IL-6(pg.mL^-1^)	10.24 ± 41.36	21.22 ± 40.13	1.047	0.299		
D-dimer(mg·L^-1^)	0.34 ± 0.51	0.40 ± 0.35	0.299	0.766		
PT (second)	10.46 ± 0.78	10.48 ± 1.10	0.074	0.941		
INR	0.89 ± 0.07	0.90 ± 0.11	0.169	0.866		
Therapeutic drugs						
Antibiotic	6 (3.5%)	1 (3.8%)	0.009	0.093		
Chinese patent medicine	133 (76.9%)	16 (61.5%)	8.827	0.093		

BMI = Body Mass Index, SpO2 = finger pulse oxygen saturation, T = temperature, ALB = albumin, WBC = white blood cell, IL-6 = Interleukin-6, PT = plasma prothrombin time, INR = International Normalized Ratio

## Discussion

Currently, Omicron has become the dominant global epidemic strain owing to its significant immune escape and higher transmissibility. The Omicron BA.5 variant has become the most prevalent Omicron subvariant worldwide [9, 21−22]; however, the characteristics of liver damage caused by the Omicron BA.5 variant remain unclear. In the study, we aim to examine and compare the clinical features, laboratory test results, and liver injury associated with the Omicron BA.5.2 patients to those of the prototype patients.

Several countries reported mild symptoms related to the Omicron strain with a mortality rate of 0.13–0.5%, which was 83–90% lower than that of the prototype and other VOC [23^–^24]. The proportion of Omicron–VOC patients with asymptomatic was 16–47.5% [25–26]. Furthermore, similar features were observed in the Omicron BA.5.2. A total of 199 the Omicron BA.5.2 patients and 157 the prototype patients were enrolled in the study. The proportion of asymptomatic Omicron BA.5.2 patients was 31%. Conversely, nearly all the prototype patients presented with various symptoms. Our previous study demonstrated that more serious lung injuries were associated with disease exacerbation, lower blood oxygen, and more serious CT manifestations in prototype patients. The hospitalized patients infected with the Omicron BA.5.2 showed little oxygen depletion, and about two-thirds had no inflammatory response on lung CT, all patients showed mild or common manifestations. The mortality rate of the prototype patients reached 7%, whereas no severe disease or death occurred in the Omicron BA.5.2 patients. This observation aligns with several studies suggesting a milder course for the Omicron. A nationwide data study in South Africa indicated that the risk of severe illness from Omicron infections reduced by 70% compared to earlier Delta infections [27]. Similarly, a retrospective cohort study (Omicron and Delta cohorts) in the United States found that proportions of hospitalization, ICU admission, and mechanical ventilation among Omicron patients were significantly reduced [28]. Interestingly, Kenrie P. Y. Hui et al. discovered that, 24 h after infection, the replication efficiency of the Omicron variant in human bronchi was 70 times higher than the prototype and Delta variant, but was 10 times lower in human lung tissue compared to the prototype [29]. This could also explain an important reason why the majority of the Omicron-infected population experiences relatively mild conditions.

In addition, the data showed that compared with the prototype, the Omicron BA.5.2 had less impact on the following parameters: WBC, PLT, HCT, PT, INR, D-dimer, and CRP. The Omicron BA.5.2 patients predominantly presented with lymphopenia, and a few patients had leukocytosis, thrombocytopenia, and hematocrit reduction. Serum CRP levels are closely related to inflammatory activity [30]. D-dimer may also reflect an inflammatory condition and predict severe and fatal cases of COVID-19 with moderate accuracy [31]. The levels of CRP and D-dimer in the Omicron BA.5.2 patients were significantly lower than in the prototype patients. Therefore, Omicron BA.5.2 patients had a lower inflammatory response than the prototype patients. Omicron BA.5.2 is thought to be less pathogenic than the prototype.

Studies have thoroughly explored the pulmonary lesions of patients with COVID-19; thus, the present study focused on liver injury. Compared with liver function indicators, the baseline levels of ALT, AST, TB, ALB, LDH, GGT, PA, and CHE as well as the proportion of those abnormal indicators were lower in the Omicron BA.5.2 patients than in the prototype patients. Specifically, the proportion of ALT, AST, TB, GGT, and LDH levels exceeding 2ULN was significantly higher in the prototype patients, these findings indicate that the Omicron BA.5.2 was associated with milder liver function impairment and a lower risk of causing liver damage than the prototype. The differences in the severity of liver damage between Omicron BA.5.2 and prototype could be associated with factors such as the severity of the disease, T, blood oxygen levels, ALB and CRP. These factors might involve the different virological characteristics of virus mutant strains, hypoxia reperfusion dysfunction, immune imbalance, and cytokine storms [16]. The foremost direct damage to the liver induced by SARS-CoV-2 is a plausible mechanism. Angiotensin-converting enzyme 2 (ACE2) has been shown to mediate SARS-CoV-2 infection, which is also expressed in cholangiocytes, hepatic sinusoidal endothelial cells (LSECs), and hepatocytes. Direct binding of the virus spike protein to ACE2 of targeted cells may result in hepatocyte and cholangiocyte injury and subsequent bile acid accumulation [16, 32−34]. Omicron variants have been associated with the prototype through multiple mutations. The specific mechanisms underlying the possible effects of different strains require further elucidation.

At present, no specific medicine exists for the treatment of SARS-CoV-2. In the early stages of the outbreak, hormones, antibiotics, arbidol, and Chinese patent medicine, among others were widely used, which may directly or indirectly cause drug-induced liver injury. However, it also has been reported that no liver injury secondary to Favipiravir was detected [35]. In a previous, authors of the current study reported that hormones were associated with liver damage [19]. Moreover, many regression studies have mentioned that the risk and proportion of liver injury were increased in patients with medium-to-large doses of glucocorticoids (≥ 10 mg/d prednisolone or equivalent drugs), antibiotics, and other drugs^[36–37]^. Only a few of the hospitalized patients infected with the Omicron BA.5.2 were treated with Chinese medicine and antipyretic drugs. Compared with early treatment, the influence of drug therapy on liver function was significantly reduced. However, although Omicron has an immune escape, the vaccine continues to have some protective effects. Even if COVID-19 vaccinations do not provide complete protection against the new variant, they will at least result in less severe infections and lower death rates [37]. Compared with the prototype patients, the Omicron BA.5.2 patients exhibited less severe inflammation and near-normal blood oxygen levels. Consequently, factors such as immune stress, inflammatory factor storms, ischemia, and hypoxia had less effect on liver damage [38]. According to the review, Omicron patients showed milder abnormal liver function.

Furthermore, this article further explored the characteristics of abnormal liver function in Omicron BA.5.2 patients. The main manifestation of liver injury was the mild elevation of AST, ALT, and GGT, which was more likely to occur in male and obese patients. Age, T, ALB, D-dimer, blood oxygen, and therapeutic drugs had little impact on liver damage. Notably, CRP was an independent factor associated with Omicron BA.5.2 patients. Ultimately, a small number of patients required liver-protective treatment. However, patients with elevated CRP still require attention.

This study has some limitations. First, it is a single-center retrospective study with a relatively small sample. Second, the assessment of liver injury is not comprehensive enough, lacking evaluation indicators such as liver biopsy puncture and radiological evaluation. Additionally, as the understanding of the virus evolves, the treatment of patients is continually adjusted. Despite these limitations, our study has successfully revealed the clinical features and liver damage characteristics of the Omicron BA.5.2 patients, and conducted comparative analysis with the prototype patients. Large-scale, multi-center clinical data are still essential to fully comprehend the impact of different SARS-CoV-2 variants on liver function.

## Conclusion

In conclusion, our study demonstrates that the Omicron BA.5.2 presents with milder symptoms and lower mortality rates compared to the prototype. The Omicron BA.5.2 patients exhibit less liver damage. Gender, BMI, and CRP may correlate with liver function impairment in the Omicron BA.5.2 patients, with CRP potentially predicting the occurrence of liver function impairment. Yet dynamic monitoring of liver function is necessary, especially among those with elevated CRP during treatment, to ensure proper management and adapt treatment strategies as needed.

## Data Availability

The datasets used and/or analyzed during the study are available from the corresponding author on reasonable request.

## Abbreviations

ALB: Albumin
ALT: Alanine transaminase
AST: Aspartate aminotransferase
ALP: Alkaline phosphatase
ACE2: Angiotensin-converting enzyme 2
BMI: Body Mass Index
COVID-19: Coronavirus disease 2019
CRP: C-reactive protein
CHE: Cholinesterase
GGT: γ-glutamyl transpeptidase
HCT: Hematocrit
INR: International Normalized Ratio
LY: Lymphocytes
LSECs: Hepatic sinusoidal endothelial cells
LDH: Lactate dehydrogenase
PLT: Platelets
PA: Prealbumin
PT: Plasma prothrombin time
SARS-CoV-2: Severe acute respiratory syndrome coronavirus 2
SpO2: Finger pulse oxygen saturation
T: Temperature
TB: Total bilirubin
ULN: Upper limit of normal
WBC: White blood cell
